# Comparative Analysis of Psychosocial Outcomes and Quality of Life Among Nulligravida, Primigravida, and Multigravida Women Diagnosed With Polycystic Ovary Syndrome (PCOS)

**DOI:** 10.7759/cureus.79895

**Published:** 2025-03-01

**Authors:** Fatima Rehman, Muhammad Muslim Khan, Ashraf LNU, Naila LNU, Rumman LNU, Shah Zeb

**Affiliations:** 1 Gynecology, Mardan Medical Complex, Medical Teaching Institution, Mardan, PAK; 2 Psychiatry, Mardan Medical Complex, Medical Teaching Institution, Bacha Khan Medical College, Mardan, PAK; 3 Research and Development, Pro-Gene Diagnostics and Research Laboratory, Mardan, PAK; 4 Obstetrics and Gynecology, Mardan Medical Complex, Medical Teaching Institution, Bacha Khan Medical College, Mardan, PAK; 5 Pharmacovigilance/Active Drug Safety Monitoring and Management, Global Fund, Combined Management Unit/Mardan Medical Complex, Medical Teaching Institution, Mardan, PAK; 6 Internal Medicine, Mardan Medical Complex, Medical Teaching Institution, Bacha Khan Medical College, Mardan, PAK

**Keywords:** nulligravida, polycystic ovary syndrome (pcos), primigravida women, psychosocial outcomes, quality of life (qol)

## Abstract

Background

Polycystic ovary syndrome (PCOS) is a prevalent endocrine disorder that significantly impacts women's physical and mental health. While its metabolic and reproductive effects are well-documented, its psychosocial impact-particularly across different reproductive stages-remains underexplored. Gravidity may influence psychological outcomes, with nulligravida women facing fertility-related distress, primigravida women experiencing pregnancy-related anxiety, and multigravida women dealing with cumulative stress and worsening PCOS symptoms. This study aims to compare anxiety, depression, stress, and quality of life (QoL) among these groups to inform targeted, stage-specific interventions that improve psychosocial well-being in women with PCOS.

Objective

This study aims to compare psychosocial well-being across nulligravida, primigravida, and multigravida women with PCOS. The findings will inform the development of targeted interventions that address the specific psychosocial needs of each reproductive group.

Methods

This cross-sectional study, conducted from July 2023 to December 2024 at Mardan Medical Complex, involved women aged 18-45 diagnosed with PCOS based on established clinical guidelines. Participants were systematically sampled and categorized by pregnancy history into nulligravida, primigravida, and multigravida groups. Validated instruments were used to assess depression, anxiety, stress, and quality of life. The data were analyzed using Kruskal-Wallis one-way ANOVA and other relevant statistical methods to compare outcomes across the groups.

Results

In this study of 510 women with PCOS, significant differences were observed across the three gravidity groups. Multigravida women had the highest mean age (32.57±4.76 years) and the highest prevalence of a family history of PCOS (40; 57.14%). Primigravida women exhibited the highest BMI (31.21±3.82) and reported the most favorable quality of life, with 56 (20.51%) rating it as Good. However, this group also had the highest levels of severe anxiety, with 211 (77.29%) reporting it. Severe depression was most prevalent among nulligravida women, with 119 (71.26%) experiencing it, while multigravida women reported the poorest quality of life, with 45 (64.29%) rating it as Poor and 28 (40%) as Very Poor. Multigravida women also had the highest stress levels, with 71 (71.43%) patients reporting high stress, and experienced the most severe PCOS symptoms, including hirsutism in 25 (35.71%) patients and acne in 37 (52.85%) patients. In comparison, severe hirsutism was observed in 49 (29.34%) of nulligravida women and 46 (16.85%) of primigravida women, while severe acne was present in 61 (36.53%) of nulligravida women and 139 (50.92%) of primigravida women.

Conclusion

These findings highlight the vital importance of personalized, stage-specific care in the effective management of PCOS, ensuring that interventions are responsive to the distinct needs and psychosocial challenges encountered by women at various reproductive stages.

## Introduction

Polycystic ovary syndrome (PCOS) is one of the most prevalent endocrine disorders affecting women of reproductive age, with an estimated global prevalence ranging from 4% to 20% depending on the diagnostic criteria used [1]. Characterized by hyperandrogenism, ovulatory dysfunction, and polycystic ovarian morphology [2], PCOS has a wide range of clinical manifestations, including menstrual irregularities, infertility, obesity, insulin resistance [3], and psychological disturbances such as anxiety, depression, and reduced quality of life (QoL) [4]. The chronic nature of PCOS and its associated complications have profound implications on the psychosocial well-being of affected individuals [5], necessitating a deeper understanding of how these factors vary across different reproductive stages, specifically among nulligravida, primigravida, and multigravida women.

PCOS is not only a medical condition but also a significant psychosocial burden for many women, affecting their emotional health, self-esteem, and social interactions [6]. The relationship between PCOS and psychosocial outcomes is complex and multifaceted, involving a combination of physiological, psychological, and social factors [7]. Women with PCOS often experience a range of psychological issues, including depression, anxiety, body image dissatisfaction, and a compromised QoL [6]. These psychological disturbances are often exacerbated by the physical symptoms of PCOS, such as hirsutism, acne, and weight gain, which can lead to significant emotional distress and social stigmatization [8].

The impact of PCOS on QoL and psychosocial well-being may differ depending on a woman’s reproductive status. Reproductive history, particularly gravidity, plays a significant role in shaping the experiences of women with PCOS. Gravidity, which refers to the number of pregnancies a woman has had, categorizes women into three groups: nulligravida (never pregnant), primigravida (pregnant for the first time), and multigravida (pregnant more than once) [9]. Each group may encounter distinct challenges and stressors related to PCOS, thereby influencing their psychosocial outcomes and QoL in unique ways.

Although the psychosocial impact of PCOS is well-documented, there is a lack of research comparing psychosocial outcomes and QoL across different reproductive stages, specifically among nulligravida, primigravida, and multigravida women. Understanding these differences is essential for developing targeted interventions that address the specific needs of each subgroup. This study aims to bridge this gap by conducting a comparative analysis of psychosocial outcomes and QoL among these three groups of women diagnosed with PCOS. By exploring the variations in psychosocial experiences across these groups, this study seeks to enhance the understanding of the psychosocial dimensions of PCOS, ultimately guiding the development of more effective, personalized interventions for women at different reproductive stages.

## Materials and methods

Study design

This cross-sectional comparative analysis study was conducted at the Mardan Medical Complex, within the Departments of Psychiatry and Gynecology from July 2023 to December 2024.

Inclusion criteria

Women included in the study were clinically diagnosed with PCOS based on the Rotterdam criteria [10] which require the presence of at least two of the following: oligo/anovulation, hyperandrogenism (clinical or biochemical), and polycystic ovaries on ultrasound. The study focused on women aged 18-45 years, a range where PCOS and its impact on psychosocial outcomes and quality of life are most prevalent. Participants were categorized into one of three groups based on their pregnancy history: nulligravida (women who had never been pregnant), primigravida (women who were pregnant for the first time), and multigravida (women who had been pregnant more than once).

Exclusion criteria

Women with other diagnosed endocrine disorders (e.g., Cushing's syndrome, thyroid disorders) that could confound the results were excluded. Women with severe psychiatric disorders such as schizophrenia, bipolar disorder, or major depressive disorder that required hospitalization were excluded, as these conditions could independently affect psychosocial outcomes and quality of life. Any woman who did not provide informed consent or withdrew from the study at any point was excluded from the analysis. Participants who did not complete the full set of questionnaires or provided incomplete responses were excluded from the final analysis.

Sample size estimation

A sample size of 510 participants was determined using the OpenEpi online sample size calculator (www.OpenEpi.com), based on a 95% confidence level and a 5% margin of error. The calculation was informed by an estimated 65% prevalence of depression and anxiety among PCOS patients, reflecting findings from previous studies on similar populations. This sample size ensures adequate power to assess the psychosocial outcomes and quality of life in the target groups [11].

Sampling technique

A systematic random sampling method was employed to select participants from the outpatient and inpatient departments of Psychiatry and Gynecology. Every third patient meeting the inclusion criteria and consenting to participate was included in the study until the desired sample size was reached for each group.

Data collection

Data collection was performed using a structured questionnaire that included standardized tools for assessing psychosocial outcomes and quality of life. These tools are described below.

Depression Assessment

Patient Health Questionnaire-9 (PHQ-9) consists of nine items, each corresponding to one of the nine Diagnostic and Statistical Manual of Mental Disorders (DSM)-IV criteria for depression. Respondents rated the frequency of symptoms over the past two weeks on a scale from 0: Not at all, 1: Several days, 2: More than half the days, and 3: Nearly every day. The total score ranges from 0 to 27, with higher scores indicating more severe depressive symptoms such as: Mild: Scores between 5-9, indicating mild depressive symptoms, Minimal: Scores between 1-4, indicating minimal or mild depression, Moderate: Scores between 10-14, indicating moderate depressive symptoms, Moderately Severe: Scores between 15-19, indicating moderately severe depression, Severe: Scores between 20-27, indicating severe depressive symptoms [12].

Anxiety Assessment

Generalized Anxiety Disorder 7 (GAD-7) consists of seven items designed to assess the severity of generalized anxiety symptoms. Respondents rated the frequency of symptoms over the past two weeks on a scale from 0: Not at all, 1: Several days, 2: More than half the days, and 3: Nearly every day. The total score ranges from 0 to 21, with higher scores indicating more severe anxiety symptoms, categorized as follows: Mild: Scores between 5-9, indicating mild anxiety, Moderate: Scores between 10-14, indicating moderate anxiety, Severe: Scores between 15-21, indicating severe anxiety [13].

Stress Assessment

Perceived Stress Scale (PSS-10) is a 10-item questionnaire that measures the perception of stress. Respondents rated how often they experienced certain feelings and thoughts during the last month on a scale from 0: Never, 1: Almost never, 2: Sometimes, 3: Fairly often, and 4: Very often. The total score ranges from 0 to 40, with higher scores indicating greater perceived stress, categorized as: Mild: Scores between 0-13, indicating mild perceived stress, Moderate: Scores between 14-26, indicating moderate perceived stress, High: Scores between 27-40, indicating high perceived stress [14].

PCOS Quality of Life Assessment

PCOS Quality of Life (PCOSQ) assessment is a disease-specific tool that evaluates the impact of PCOS on various aspects of life, including emotional well-being, body hair growth, weight, infertility, and menstrual issues. Respondents rated their experiences on a scale from 1: Always, to 7: Never. The total score was calculated by averaging the domain scores, with higher scores indicating a better quality of life. The scores are categorized as: Good: High scores indicating a positive quality of life, Moderate: Scores reflecting an average quality of life, Poor: Scores suggesting a low quality of life, Very Poor: Low scores indicating a severely impacted quality of life [15].

Participants in the study were interviewed by trained research assistants, and doctors who ensured accurate completion of all questionnaire sections. Data collected included demographic information, obstetric history, current comorbidities, and PCOS-related symptoms, which were categorized based on severity. Hirsutism was classified into mild (light, fine hair), moderate (noticeable coarse hair on the face, chest, or abdomen), and severe (extensive, thick male-pattern hair growth). Acne and oily skin were categorized as mild (occasional breakouts), moderate (frequent acne requiring over-the-counter treatment), and severe (persistent cystic acne requiring prescription treatment). Weight gain was classified as mild (slight weight gain), moderate (significant weight gain difficult to control), and severe (obesity with metabolic issues like insulin resistance or type 2 diabetes). Thinning hair or male-pattern baldness was categorized into mild (slight thinning), moderate (noticeable thinning and widening of the scalp part), and severe (significant hair loss resembling male-pattern baldness). Darkening of the skin (acanthosis nigricans) was categorized as mild (slight darkening in body folds), moderate (noticeable dark patches in areas like the neck, armpits, and groin), and severe (extensive, dark patches causing discomfort or itching). This comprehensive assessment helped in understanding the severity of symptoms among participants.

Data analysis

Continuous variables were summarized using the mean and standard deviation, while categorical variables were described with frequencies and percentages. To assess differences among the Nulligravida, Primigravida, and Multigravida groups across all variables, the Kruskal-Wallis one-way analysis of variance (ANOVA) test was utilized. To further investigate the relationships among variables, an interconnected relationship map was developed, facilitating the identification of strong associations between specific factors and the respective groups. Density plots were generated to visually assess the distribution of responses for each question related to depression, anxiety, stress, and quality of life across the different groups. Additionally, coefficient plots derived from logistic regression analyses were employed to evaluate the strength and direction of the relationship between each question and the overall outcomes related to depression, anxiety, stress, and quality of life within the groups. A p-value of less than 0.05 was considered statistically significant. All statistical analyses were conducted using SPSS software (version 29.0, IBM Corp., Armonk, NY, USA) and R (version 4.4.2, R Foundation for Statistical Computing, Vienna, Austria).

Ethical considerations

The study protocol was approved by the Institutional Review Board (IRB) No: 557/BKMC of the hospital where the study was conducted. All participants provided written informed consent prior to enrollment. Confidentiality of patient data was ensured by assigning unique identification numbers to each participant. Data were stored securely and were accessible only to the study investigators.

## Results

The study examined 510 women with PCOS, categorized as nulligravida (167 patients, 32.75%), primigravida (273 patients, 53.53%), and multigravida (70 patients, 13.73%). Multigravida women were older (32.57 years), had the longest duration of PCOS diagnosis (7.35 years), and the highest prevalence of family history (40 patients, 57.14%). Primigravida women had the highest BMI (31.21 ± 3.82). Multigravida women (30 patients, 42.86%) were more likely to be educated and were predominantly housewives (60 patients, 85.71%). Fertility treatment was most common among multigravida women (55 patients, 78.57%) (Table 1).

**Table 1:** Demographic and Clinical Characteristics of Nulligravida, Primigravida, and Multigravida Women With Polycystic Ovary Syndrome (PCOS) Data is presented as frequency and percentage or as median and inter quartile range. P value with *** represent no statistics were computed because the characteristic is either present in 100% or 0% of the population. P value <0.05 is statically significant

Characteristics	All patients	Nulligravida	Primigravida	Multigravida	P values
	510(100)	167(32.75)	273(53.53)	70(13.73)	***
Age	28.37 ± 6.35	27.75 ± 6.77	27.67 ± 6.37	32.57 ± 4.76	0.000
BMI	30.28 ± 4.57	29.64 ± 5.02	31.21 ± 3.82	30.28 ± 4.57	0.038
Family History of PCOS	210 (41.18)	73 (43.71)	97 (35.53)	40 (57.14)	0.003
Duration of PCOS diagnosis	6.44 ± 4.55	3.39 ± 2.33	6.02 ± 5.91	7.35 ± 6.22	0.048
Educational level					
Educated	150 (29.41)	50 (29.94)	70 (25.64)	30 (42.86)	0.018
Uneducated	360 (70.59)	117 (70.06)	203 (74.36)	40 (57.14)	
Employment status					
House Wife	370 (72.55)	100 (59.88)	210 (76.92)	60 (85.71)	0.000
Job	50 (9.80)	28 (16.77)	17 (6.23)	5 (7.14)	
Jobless	90 (17.65)	39 (23.35)	46 (16.85)	5 (7.14)	
Current pregnancy status					
Gestational age					
1st trimester	166 (32.54)	0.00 (0.00)	143 (52.38)	23 (32.85)	0.00
2nd trimester	103 (20.19)	0.00 (0.00)	69 (25.27)	34 (48.57)	
3rd trimester	74 (14.50)	0.00 (0.00)	61 (22.34)	13 (18.57)	
Gravidity	3.49 ± 2.38	0.00 ± 0.00	0.00 ± 0.00	3.49 ± 2.38	***
Parity	2.98 ± 2.35	0.00 ± 0.00	0.00 ± 0.00	2.98 ± 2.35	***
Type of conception used					
Fertility treatment	315 (61.76)	89 (53.29)	171 (62.64)	55 (78.57)	0.049
Natural	195(38.23)	78(46.71)	102 (37.36)	15 (21.43)	

The analysis of PCOS symptoms and comorbidities among nulligravida, primigravida, and multigravida women reveals several significant findings. Irregular menstrual cycles were similarly prevalent across groups (p = 0.580). Severe hirsutism was more common in multigravida women (35.71%) compared to nulligravida (29.34%) and primigravida (16.85%) (p=0.050). Severe acne and oily skin were more frequent in multigravida women (52.85%) (p=0.043), while severe weight gain was more common in nulligravida women (44.31%) (p=0.048). Thinning hair was most severe in primigravida women (64.29%) (p=0.031), and darkening of the skin was more severe in multigravida women (64.29%) (p=0.008). Obesity was significantly higher in multigravida women (71.43%) (p=0.028), whereas fibroids were more common in nulligravida women (20.36%) (p=0.041). Other comorbidities showed no significant differences (Table 2).

**Table 2:** Polycystic Ovary Syndrome (PCOS) Symptoms and Comorbidities Among Nulligravida, Primigravida, and Multigravida Women Data is presented as frequency and percentage. P value with *** represent no statistics were computed because the characteristic is either present in 100% or 0% of the population. P value <0.05 is statically significant DM: diabetes mellitus

Characteristics	All patients	Nulligravida	Primigravida	Multigravida	P values
	510(100)	167(32.75)	273(53.53)	70(13.73)	***
PCOS symptoms					
Irregular Menstrual Cycles					
Mild	50 (9.80)	12 (7.19)	23 (8.42)	15 (21.43)	0.580
Moderate	405 (79.41)	145 (86.83)	210 (76.92)	50 (71.43)	
Severe	55 (10.78)	10 (5.99)	40 (14.65)	5 (7.14)	
Hirsutism					
Mild	90 (17.65)	30 (17.96)	45 (16.48)	15 (21.43)	0.050
Moderate	300 (58.82)	88 (52.69)	182 (66.67)	30 (42.86)	
Severe	120 (23.53)	49 (29.34)	46 (16.85)	25 (35.71)	
Acne and Oily Skin					
Mild	100 (19.61)	37 (22.16)	43 (15.75)	20 (28.57)	0.043
Moderate	173 (33.92)	69 (41.32)	91 (33.33)	13 (18.57)	
Severe	237(46.47)	61 (36.53)	139 (50.92)	37 (52.85)	
Weight Gain and Difficulty Losing Weight					
Mild	70 (13.73)	26 (15.57)	29 (10.62)	15 (21.43)	0.048
Moderate	235 (46.08)	67 (40.12)	138 (50.55)	30 (42.86)	
Severe	205 (40.20)	74 (44.31)	106 (38.83)	25 (35.71)	
Thinning Hair or Male-Pattern Baldness:					
Mild	141 (27.65)	56 (33.53)	74 (27.11)	10 (14.29)	0.031
Moderate	240 (47.06)	64 (38.32)	130 (47.62)	45 (64.29)	
Severe	130 (25.49)	46 (27.54)	69 (25.27)	15 (21.43)	
Darkening of the Skin (Acanthosis Nigricans)					
Mild	214 (41.96)	57 (34.13)	113 (41.39)	45 (64.29)	0.008
Moderate	165 (32.35)	61 (36.53)	89 (32.60)	15 (21.43)	
Severe	130 (25.49)	49 (29.34)	71 (25.91)	10 (14.29)	
Comorbidities					
Anemia	250 (49.02)	82 (49.10)	131 (48.00)	37 (52.86)	0.768
Obesity	295 (57.84)	88 (52.69)	157 (57.51)	50 (71.43)	0.028
Fibroid	81 (15.88)	34 (20.36)	34 (12.45)	13 (18.57)	0.041
Ovarian cyst	402 (78.82)	127 (76.05)	223 (81.68)	52 (74.29)	0.227
Endometriosis	61 (11.96)	21 (12.57)	35 (12.82)	5 (7.14)	0.409
Hypertension	159 (31.18)	49 (29.34)	90 (32.97)	20 (28.57)	0.642
T1 DM	11 (2.16)	4 (2.40)	5 (1.83)	2 (2.86)	0.843
T2 DM	119 (23.33)	42 (25.15)	60 (21.98)	17 (24.29)	0.733

The analysis of PCOS-related quality of life, stress, depression, and anxiety among nulligravida, primigravida, and multigravida women reveals significant variations. Primigravida women reported the highest quality of life scores (20.51%) compared to nulligravida (12.57%) and multigravida (12.86%) (p=0.024). Conversely, multigravida women experienced the poorest quality of life, with higher rates of poor (64.29%) and very poor (40.00%) assessments. Stress levels were uniformly high across groups, with multigravida women reporting the highest levels of stress (71.43%) (p=0.002). Depression was most severe among nulligravida women (71.26%) (p=0.037), while primigravida women exhibited the highest prevalence of severe anxiety (77.29%) (p=0.041) (Table 3).

**Table 3:** Severity of Quality of Life, Stress, Depression, and Anxiety in Nulligravida, Primigravida, and Multigravida Women With Polycystic Ovary Syndrome (PCOS) Data is presented as frequency and percentage. P value with *** represent no statistics were computed because the characteristic is either present in 100% or 0% of the population. P value <0.05 is statically significant

Characteristics	All patients	Nulligravida	Primigravida	Multigravida	P values
	510(100)	167(32.75)	273(53.53)	70(13.73)	***
Overall PCOS Quality of Life Assessment					
Good	86(16.86)	21(12.57)	56(20.51)	09(12.86)	0.024
Moderate	118(23.14)	38(22.75)	59(21.61)	21(30.00)	
Poor	146(28.63)	45(26.95)	12(4.40)	45(64.29)	
Very poor	160(31.37)	69(41.32)	69(25.27)	28(40.00)	
Overall Stress Assessment: Perceived Stress Scale (PSS-10)					
Mild	00(0.00)	00(0.00)	00(0.00)	00(0.00)	0.002
Moderate	194(38.04)	67(40.12)	107(39.19)	20(28.57)	
High	316(61.96)	100(59.88)	166(60.81)	50(71.43)	
Overall Depression Assessment: Patient Health Questionnaire-9 (PHQ-9)					
Mild	25(4.90)	14(8.38)	11(4.03)	00(0.00)	0.037
Minimal	05(0.98)	00(0.00)	00(0.00)	05(7.14)	
Moderate	50(9.80)	13(7.78)	22(8.06)	15(21.43)	
Moderately severe	95(18.63)	21(12.57)	64(23.44)	10(14.29)	
Severe	335(65.69)	119(71.26)	176(64.47)	40(57.14)	
Overall Anxiety Assessment: Generalized Anxiety Disorder 7					
Mild	50(9.80)	21(12.57)	19(9.96)	10(14.29)	0.041
Moderate	80(15.69)	27(16.17)	43(15.75)	10(14.29)	
Severe	380(74.51)	119(71.26)	211(77.29)	50(71.43)	

**Figure 1:**
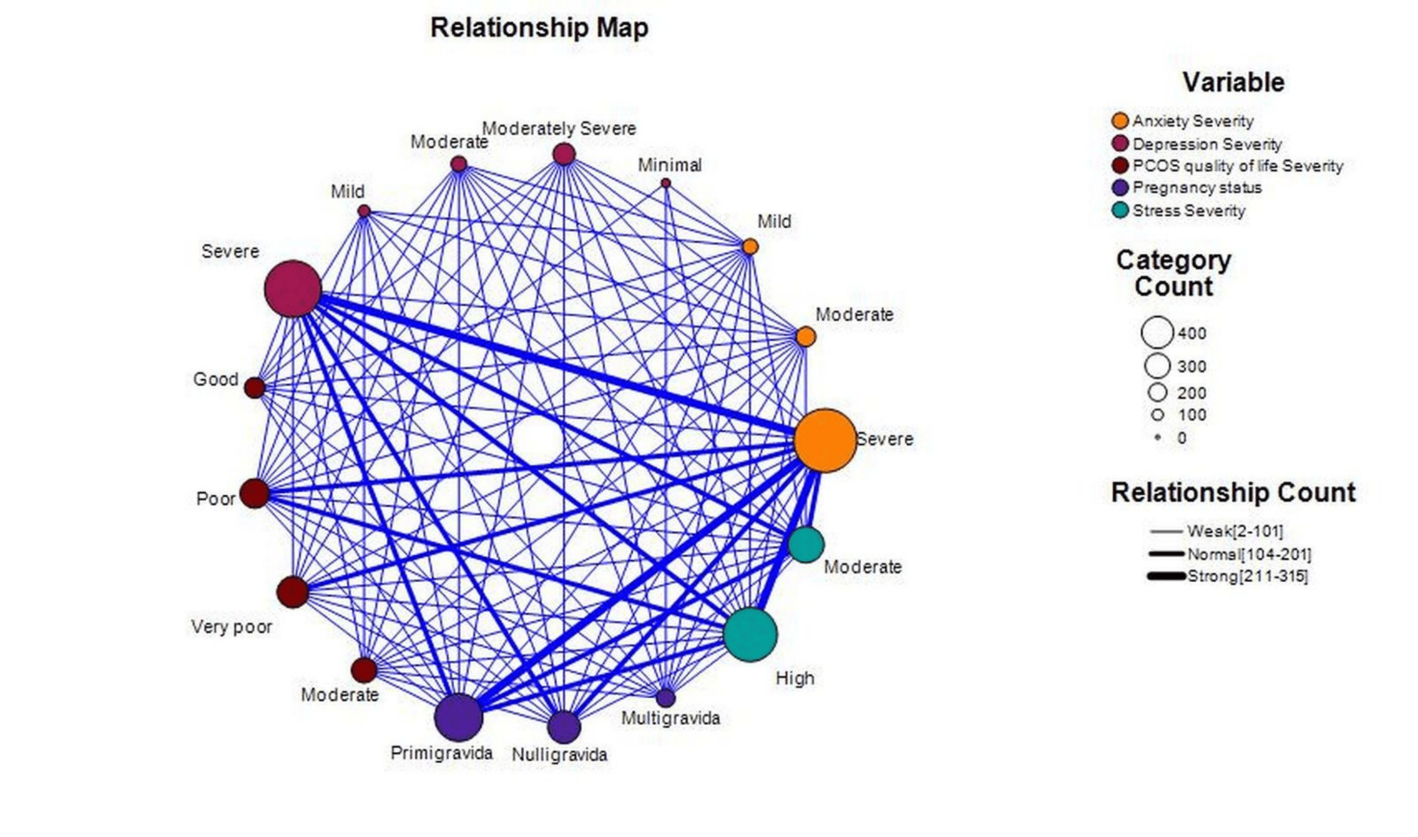
Interconnections of Anxiety, Stress, Depression, and Quality of Life in Women With Polycystic Ovary Syndrome (PCOS): A Gravidity-Based Relationship Map

The relationship map reveals significant associations between psychosocial outcomes and quality of life in nulligravida, primigravida, and multigravida women with PCOS. Primigravida women are strongly linked to severe anxiety, while nulligravida women are more prone to severe depression. Multigravida women are notably associated with poor quality of life. The map highlights that severe anxiety and depression are closely related to poor quality of life across all groups. High stress levels, particularly in multigravida women, exacerbate anxiety and depression, creating a cyclical impact on quality of life. Moderate anxiety and depression also show notable associations, especially in primigravida women, indicating varied impacts based on pregnancy history (Figure 1).

**Figure 2:**
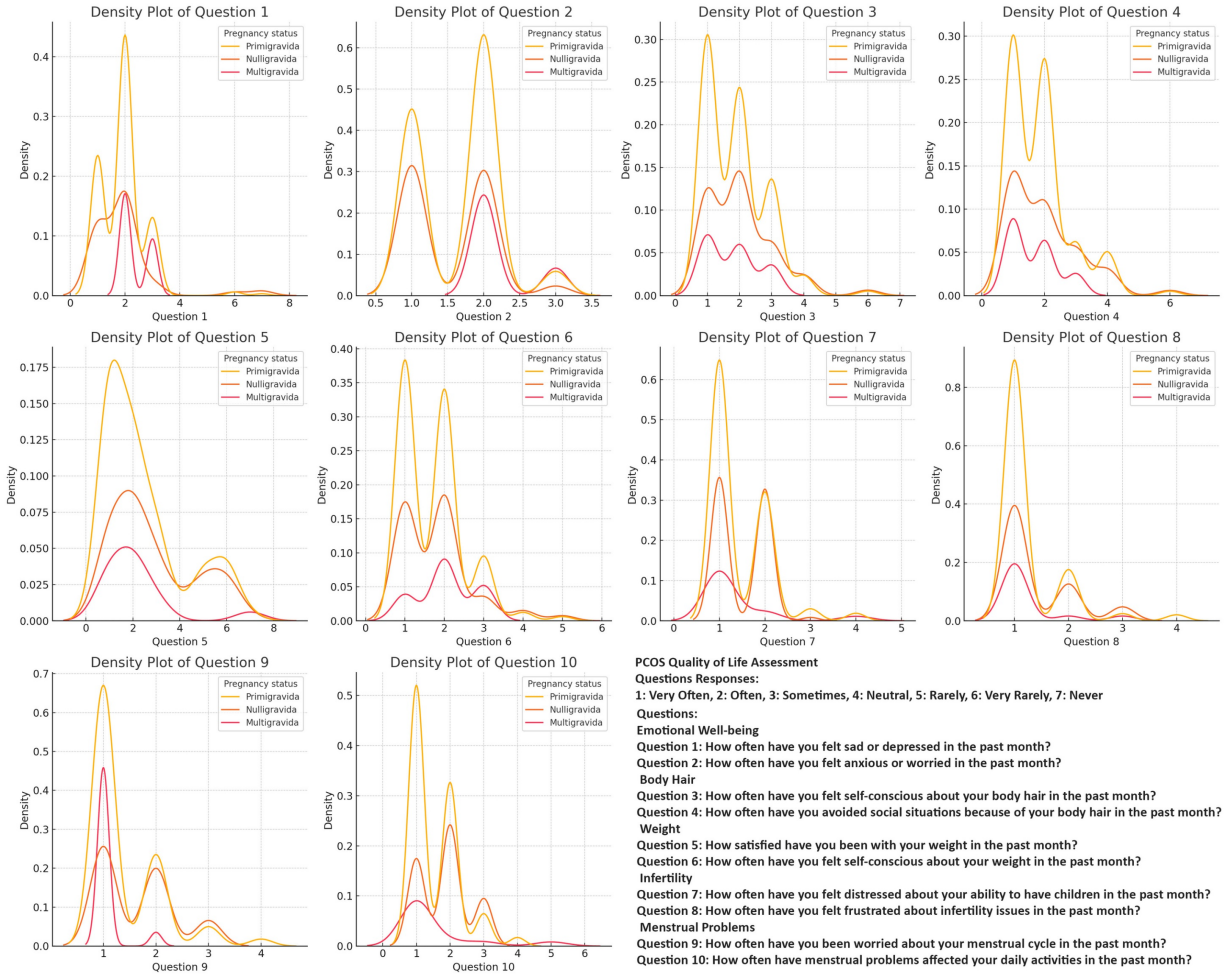
Comparing Polycystic Ovary Syndrome (PCOS) Quality of life Severity in PCOS: Question Patterns Among Nulligravida, Primigravida, and Multigravida Women

**Figure 3:**
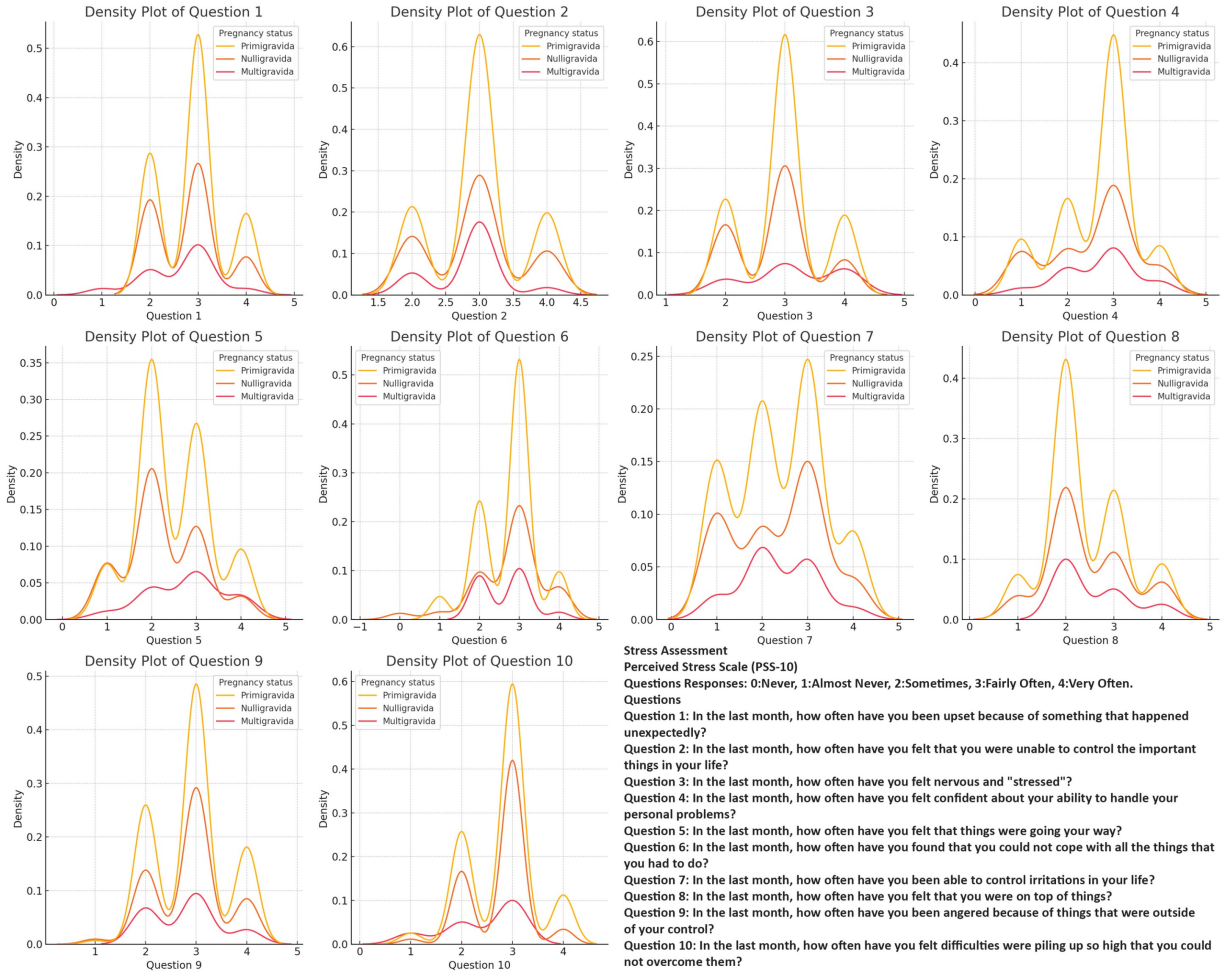
Comparing Stress Severity in Polycystic Ovary Syndrome (PCOS): Question Patterns Among Nulligravida, Primigravida, and Multigravida Women

**Figure 4:**
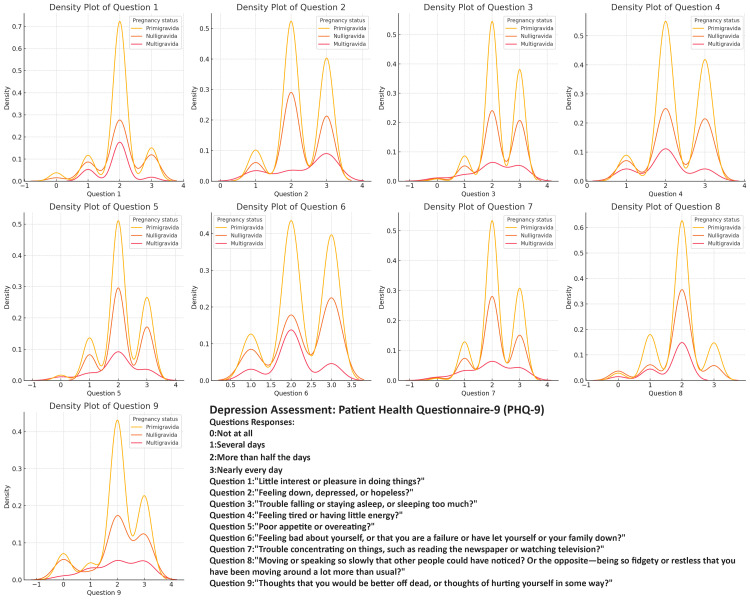
Comparing Depression Severity in Polycystic Ovary Syndrome (PCOS): Question Patterns Among Nulligravida, Primigravida, and Multigravida Women

**Figure 5:**
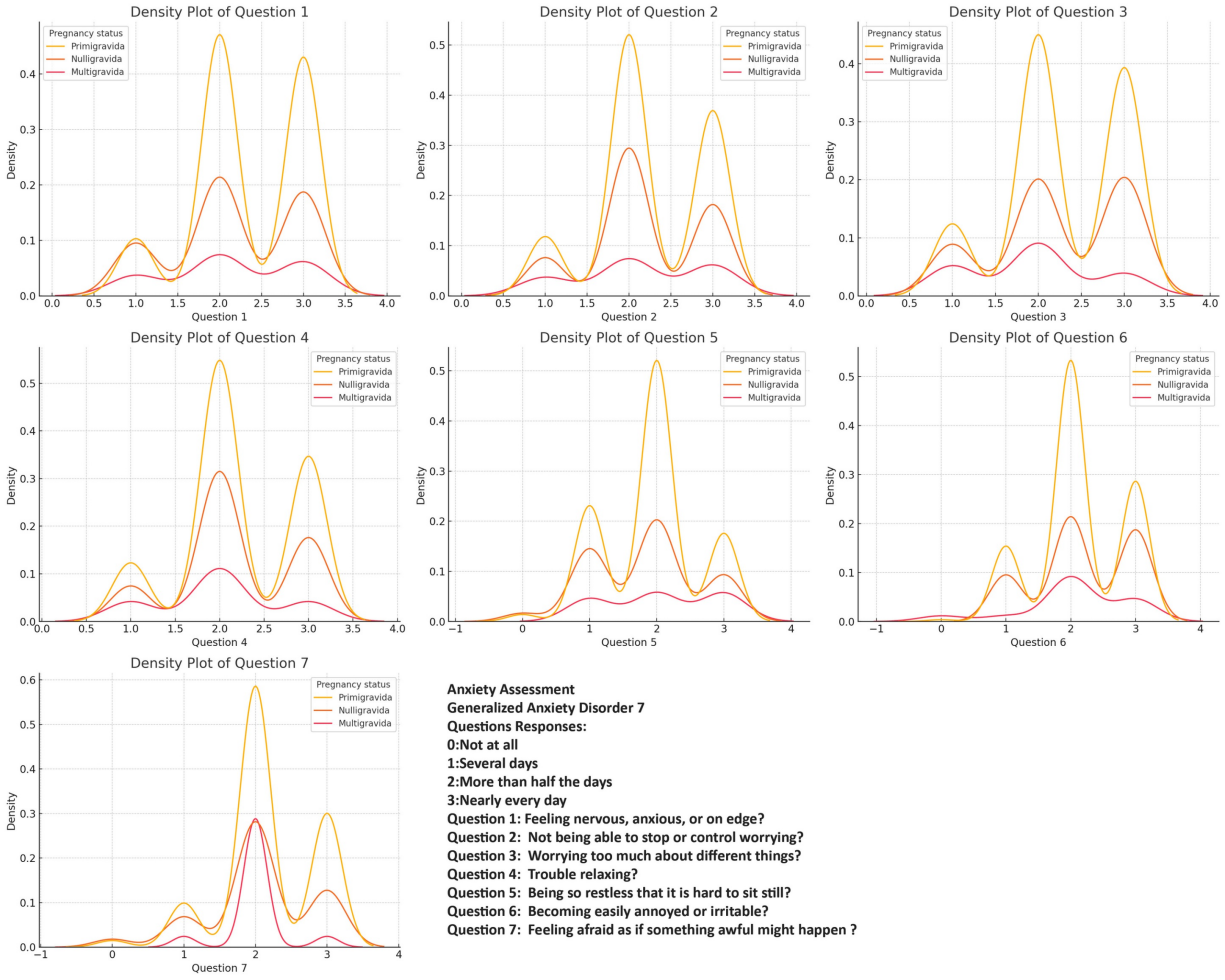
Comparing Anxiety Severity in Polycystic Ovary Syndrome (PCOS): Question Patterns Among Nulligravida, Primigravida, and Multigravida Women

Across all the assessments (anxiety, depression, quality of life, and stress), primigravida respondents consistently exhibit the highest and most concentrated density peaks, indicating that they experience more uniform and moderate levels of anxiety, depression, and stress, as well as challenges related to PCOS. Nulligravida respondents show a similar pattern but with slightly less intensity and consistency, reflecting a moderate experience of these challenges with some variability. In contrast, multigravida respondents display a broader range of responses across all variables, with lower and more dispersed density peaks (Figures 2-5).

**Figure 6:**
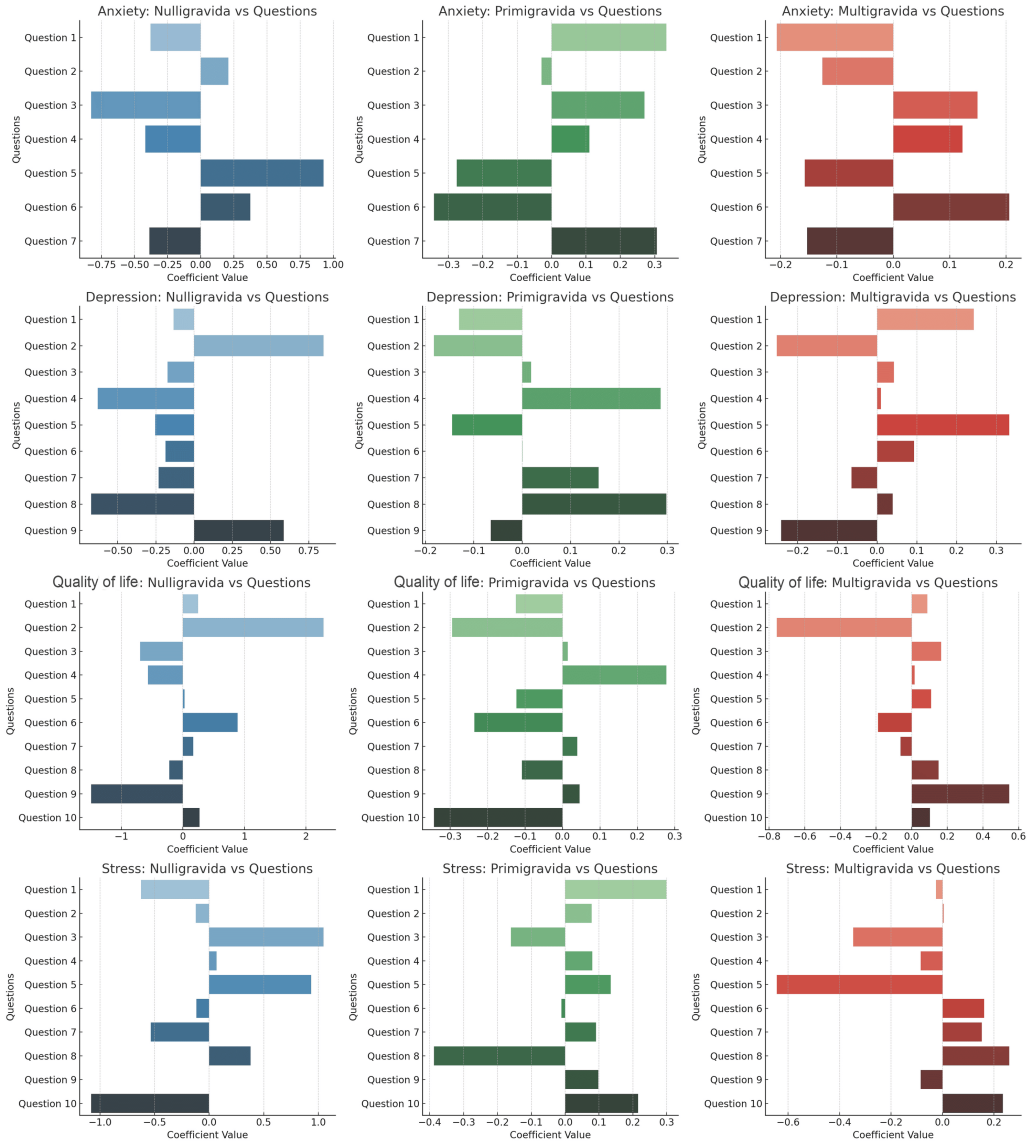
Comparative Coefficient Analysis of Anxiety, Depression, Stress, and Quality of Life in Nulligravida, Primigravida, and Multigravida Women With Polycystic Ovary Syndrome (PCOS)

The coefficient value charts analyze the relationships between anxiety, depression, PCOS-related quality of life, and stress among nulligravida, primigravida, and multigravida women with PCOS. For anxiety, nulligravida women are most affected by nervousness (Question 1) and restlessness (Question 5), while primigravida women show heightened anxiety related to excessive worrying (Question 3) and restlessness. Multigravida women experience significant anxiety from nervousness and fears of something terrible happening (Questions 1 and 7). Regarding depression, nulligravida women are impacted by feeling down and moving slowly (Questions 2 and 8), primigravida women struggle with sleep issues and negative self-view (Questions 3 and 6), and multigravida women are most affected by lack of interest in activities and feeling down (Questions 1 and 2). For PCOS-related quality of life, nulligravida women are most negatively affected by dissatisfaction with weight and menstrual issues (Questions 6 and 10), primigravida women by body hair concerns and fertility worries (Questions 3 and 7), and multigravida women by sadness and weight dissatisfaction (Questions 1 and 5). In terms of stress, nulligravida women report high stress related to nervousness and confidence (Questions 4 and 5), primigravida women are most stressed by unexpected events and problem management (Questions 1 and 5), and multigravida women face the highest stress from feeling overwhelmed and a lack of control (Questions 6 and 8) (Figure 6).

## Discussion

The current study provides a comprehensive examination of the demographic and clinical characteristics, symptoms, comorbidities, and psychosocial outcomes associated with PCOS among nulligravida, primigravida, and multigravida women. The findings reveal significant differences across these groups, underscoring the influence of gravidity on the overall experience and health outcomes in women with PCOS.

The higher Body Mass Index (BMI) observed among primigravida women, compared to nulligravida and multigravida women, aligns with previous studies indicating a link between PCOS and increased weight gain, particularly in first-time pregnant women [16]. The age variations among these groups are consistent with existing literature, which highlights age as a significant factor associated with increased parity and the duration of PCOS diagnosis [17]. Moreover, age and BMI are critical factors influencing in vitro fertilization outcomes, emphasizing the need to consider these variables in reproductive interventions for women with PCOS [18]. Additionally, age has been identified as a predictor of hypertensive disorders during pregnancy, further underscoring its impact on such conditions in women with PCOS [19].

The study also reports a higher prevalence of a family history of PCOS among multigravida women, suggesting a potential genetic predisposition that becomes more apparent with successive pregnancies. This observation is supported by previous research indicating that a family history of PCOS significantly increases the likelihood of developing the syndrome [20]. Furthermore, the longer duration of PCOS diagnosis in multigravida women highlights the chronic nature of the condition and its cumulative impact over time [21].

Differences in educational level and employment status among the gravidity groups suggest that socioeconomic factors significantly influence reproductive decisions and health outcomes in women with PCOS. The higher proportion of educated multigravida women might indicate that education influences family planning decisions, potentially leading to delayed pregnancies. Conversely, the higher employment rate among nulligravida women could reflect the challenges of balancing work and family life, contributing to delayed childbearing. These findings underscore the complex interplay between education, employment, and reproductive choices in women with PCOS [22].

The study further identifies a higher prevalence of severe hirsutism and acne among multigravida women, implying a potential worsening of these symptoms with subsequent pregnancies or over time, a pattern supported by existing research [23]. Additionally, weight gain and difficulty losing weight were more pronounced in nulligravida women, possibly due to the absence of metabolic shifts associated with pregnancy. However, the high prevalence of obesity among multigravida women suggests that weight management becomes increasingly challenging with each pregnancy [24]. Primigravida women exhibited a higher prevalence of hair thinning or male-pattern baldness, likely due to hormonal fluctuations during pregnancy, while multigravida women showed a higher prevalence of acanthosis nigricans, possibly indicating more severe insulin resistance exacerbated by repeated pregnancies [25].

Regarding comorbidities, the study highlights the higher prevalence of obesity among multigravida women and fibroids among nulligravida women, underscoring the complex relationship between reproductive history and additional health conditions. Obesity has been identified as a significant risk factor for developing comorbidities in PCOS, including metabolic syndrome and type 2 diabetes, while hormonal imbalances associated with PCOS may contribute to the development of fibroids in nulligravida women [26,27].

The study's findings on psychosocial outcomes reveal differential impacts of PCOS on quality of life, stress, depression, and anxiety across gravidity groups. Our study found that multigravida women with PCOS experience significantly poorer quality of life compared to nulligravida and primigravida women. This aligns with existing literature indicating that repeated pregnancies can exacerbate the negative effects of PCOS on well-being due to challenges such as weight gain, hormonal fluctuations, and the added responsibilities of childcare [28,29]. Stress levels were highest among multigravida women, suggesting that managing multiple pregnancies and children, combined with PCOS symptoms, may contribute to higher stress levels [28]. Additionally, the severity of depression was most pronounced in nulligravida women, possibly reflecting the psychological burden associated with infertility or delayed childbearing [30,31]. Primigravida women, on the other hand, were more likely to experience severe anxiety, particularly related to pregnancy and future reproductive potential, consistent with previous studies highlighting anxiety as a prevalent comorbidity in women with PCOS, especially during pregnancy [32,33].

The density plots of the PCOS Quality of Life Assessment, Perceived Stress Scale (PSS), PHQ-9, and GAD-7 responses among different gravidity groups offer valuable insights into the distribution of experiences among women with PCOS. Primigravida women consistently reported higher frequencies of negative experiences, particularly regarding anxiety, depression, and stress, suggesting that the combination of the first pregnancy experience and chronic PCOS symptoms contributes to a more severe psychosocial burden in this group. Noteworthy peaks in specific questions related to body image concerns and worries about menstrual cycles indicate that these issues are prevalent across all gravidity groups and should not be overlooked in clinical practice [34,35].

A particularly important finding, not widely explored in the literature, is the relationship map illustrating the complex interplay between anxiety, depression, stress, and quality of life in women with PCOS. The strong associations between severe anxiety in primigravida women and severe depression in nulligravida women suggest that these psychosocial factors are deeply interconnected and may mutually reinforce each other. For example, high anxiety levels may exacerbate depressive symptoms, leading to a decline in overall quality of life. This interconnectedness underscores the importance of a holistic approach to managing PCOS that addresses both physical symptoms and the mental and emotional well-being of the patient.

While this study offers valuable insights into the comparative analysis of psychosocial outcomes and quality of life among nulligravida, primigravida, and multigravida women with PCOS, it has several limitations. First, the study's single-center design may limit its external validity, making the findings less generalizable to women with PCOS in different geographic regions or healthcare settings. Second, the cross-sectional design precludes the establishment of causal relationships between reproductive status and psychosocial well-being. Third, excluding women with severe psychiatric disorders may underestimate the true psychosocial burden among women with PCOS. Fourth, systematic random sampling may introduce selection bias, as women seeking treatment at the medical complex might differ significantly from those who do not. Fifth, the study does not fully account for differences in treatment regimens, which could confound the results. Sixth, the reliance on assessments conducted within a specific timeframe might not capture the long-term psychosocial effects of PCOS. Finally, focusing primarily on gravidity status may overlook other important factors, such as marital status, social support, and coping mechanisms, which could also influence psychosocial outcomes and quality of life in this population. These limitations should be considered when interpreting the findings and designing future research.

## Conclusions

The strong associations between anxiety, depression, stress, and quality of life across all gravidity groups underscore the interconnectedness of psychosocial factors in women with PCOS. A holistic approach to managing PCOS that addresses both physical symptoms and mental health is crucial. Tailored interventions that consider the specific needs of nulligravida, primigravida, and multigravida women can improve overall well-being and quality of life in this population.
